# Factors influencing the adoption of generative artificial intelligence into classroom teaching by university teachers: An empirical study using SPSS PROCESS macros

**DOI:** 10.1371/journal.pone.0324875

**Published:** 2025-08-20

**Authors:** Yong Xiang, Chenxin Yang, Zhigang Jin, Wanshu Zhao

**Affiliations:** School of Civil Engineering, Architecture, Environment, Xihua University, Chengdu, China; University of Tartu, ESTONIA

## Abstract

With the development of science and technology, higher education faces the challenge of AIGC. As one of the central bodies of higher education, it is essential to understand whether teachers accept this new technology and what factors influence their choice. This study collected survey data from teachers at 42 universities in China and constructed a structural equation model based on social cognitive theory to explore the factors influencing the adoption of AIGC by university teachers. In this study, partial least squares (PLS) were used to analyze the validity and reliability of the data and process macro model 4 and model 61 based on SPSS software were used to verify the mechanism of the influence of each factor in the structural equation modeling on willingness to choose. It was found that self-efficacy is a key factor in the willing to choose from the perspective of college teachers; the positive influence of self-efficacy on willingness to choose is more significant when the level of outcome expectation is higher; external environmental factors will strengthen the positive influence of self-efficacy on outcome expectation and willing to choose; and the ability of self-improvement will also enhance the positive influence of self-efficacy on outcome expectation. This study provides an in-depth exploration of the critical factors influencing teachers’ adoption of AIGC, providing valuable insights and empirical evidence for decision-making on technology integration in education and providing educational administrators and policymakers with references on how to promote teachers’ adoption of new technologies.

## Introduction

Generative Artificial Intelligence, abbreviated as AIGC(Artificial Intelligence Generated Content), has swept the world, opening a new generation of technological revolutions. The powerful capabilities of AIGC have attracted the attention of education experts, as it seems to have changed entirely existing educational practices. Universities are exploring its vast potential for future teaching and learning [1,2]. To encourage the process of AIGC entering universities, governments have continually implemented AIGC education policies [3]. However, most education industry practitioners cannot fully utilize AIGC in teaching. Research shows that AIGC has the potential to significantly improve teaching efficiency, with some studies showing an increase of up to 96% [4]. By automating the generation of course content and student assessments through AIGC, teachers can save much time to focus on improving the quality of classroom teaching and student engagement [5]. AIGC can help achieve inclusive education, ensuring students receive a high-quality education regardless of their family background or individual abilities. Through a customized learning experience, AIGC can help students with different needs acquire knowledge easily and happily during the learning process [6]. However, the primary focus of current relevant research is on the impact of AI on student achievement and experience [7]. Therefore, it is urgent to investigate the factors that affect teachers’ adoption of AIGC, laying the foundation for guiding teachers to use AIGC in the future.

A common description of AIGC is that it is a technology that creates new and unique content using machine learning models, including text, images, and music. Sabzalieva and Valentini [8] showed that one tool that might completely alter traditional educational institutions is AIGC. AIGC can ensure that people from different family backgrounds and with varying learning abilities can receive a high-quality education. In addition, education can take on various forms, from one-on-one tutoring to supporting effective student teamwork [9].

The emergence of AIGC saves teachers time preparing lessons, reduces the workload of correcting homework, and can summarize each student’s knowledge gaps [10]. Glover and Miller [11] believe that if teachers are not aware of the need always to adopt a learning attitude in teaching, they may have problems using new things and have a limited impact on learning and teaching. The internal factors that affect teachers’ choice to use new technologies include the teacher’s age, gender, years of teaching experience, the teacher’s central, the subject they teach, and the teacher’s learning ability. Teachers’ self-efficacy determines their sensitivity and adaptability when using new technologies. Teachers who can integrate new technologies into their teaching will improve their sense of efficacy. However, there is no research on teachers’ self-efficacy as a factor affecting teachers’ acceptance of technology; therefore, in this paper, the self-efficacy enhancement of college teachers is considered one of the factors affecting the adoption of AIGC to classroom education by college teachers.

Teachers’ self-perception of their teaching talents is the foundation of their self-efficacy [12]. It is a crucial factor affecting teachers’ motivation to teach and positively correlates with high-quality teaching [13]. Some studies have shown that AIGC can provide teaching feedback and assessment [14] to help teachers reflect on their teaching processes and strategies, prompting them to clarify their teaching objectives and progress [15], adjust teaching strategies promptly, and improve their teaching ability and effectiveness, thereby achieving a sense of self-efficacy [16]. After teachers master AIGC technology, they can use the teaching resources generated by AIGC to create more innovative and interactive classrooms, thereby increasing student engagement in the school [17]. This successful experience, in turn, enhances teachers’ sense of self-efficacy.

College students generally have a positive attitude toward the potential of AIGC technology in teaching and learning [18]. Studies have found that primary and secondary school students may not accept new technologies and require more guidance and Training [19]. Compared to primary and secondary school students, college students have more exposure to computers and generally have a higher level of computer literacy and critical thinking [20], which allows them to understand and use the content generated by AIGC more deeply. College teachers adopt AIGC into the classroom, and students are more receptive to the changes this technology brings, promoting interaction between teachers and students and enhancing students’ ability to learn independently and think creatively [21]. Although there are many studies on the use of new technologies in education, they are not targeted to differentiate the age of the students or to consider the students’ receptivity to AIGC, so this study is only focused on students in higher education and on influencing teachers in higher education to adopt new technologies to classroom education.

With the advancement of technology, more and more researchers have begun to explore technology integration in education. For example, Graham, Ogbonnaya [22] examined teachers’ intention to incorporate ICT technologies into T&L based on the TAM model, and Ivanov, Soliman [23]drew on the TPB model to extract variables related to GenAI and verified its effect on teachers’ and students’ intentions to use GenAI. However, traditional adoption intention modeling theories, such as the Technology Acceptance Model (TAM) and the Theory of Planned Behavior (TPB), while having some explanatory power in explaining technology adoption behaviors, focus mainly on teachers’ psychological factors and less on the interactions between environmental factors and teachers’ behaviors. Based on social cognitive theories, Zhang and Qian [24] explored the relationship between social support and the correlation between adolescents’ academic performance. Although a more comprehensive framework was used to explore adolescent academic achievement, less attention was paid to educators. It can be seen that social cognitive theory provides a more comprehensive framework that integrates the interplay of individual, environmental, and behavioral factors. The theory offers a more thorough explanation of the mechanisms by which higher education teachers adopt AIGC techniques. Rahmati [24] explored the role of pronunciation on teaching practices in terms of institutional and socio-cultural factors of practice change based on social cognitive theory. Based on this line of thinking, this study incorporated AIGC and individual, environmental, and behavioral-related factors as key variables in the theoretical model to further enrich higher education teachers’ understanding of the process of AIGC technology adoption.

This study offers several contributions. First, it provides a new perspective on technology-integrated education by using social cognitive theory to identify the drivers that affect college teachers’ adoption of AIGC in the classroom. Second, using social cognitive theory as a starting point to explore the factors influencing college teachers’ use of AIGC from three dimensions: environmental, personal, and behavioral; this multidimensional analysis helps to fully understand the motivations, barriers, and behavioral patterns of college teachers in adopting AIGC technology. In conclusion, AIGC as an emerging technology has initiated changes in several fields globally, but its decision-making process in higher education scenarios, especially regarding whether college teachers introduce it into classroom teaching or not, has been relatively understudied. This study not only fills the theoretical gap in the application of AIGC in the field of higher education but also provides new ideas and methods for the deep integration and synergistic development of college teachers and AIGC, which helps to promote the development of the education field in the direction of intelligence, personalization, and efficiency. In addition, the findings of this study will help college and university teachers to enhance their teaching effectiveness and improve their professional competence; help educational administrators to promote academic innovation and establish a supportive environment; and help policymakers to rationally allocate educational resources and formulate long-term educational technology planning and so on.

As the research progresses, it is critical to review the detailed literature on the merits of AIGC for each domain and the impact of factors contained in social cognitive theory on teacher instruction. The second part of the article presents the theoretical framework and research hypotheses, and the third part of the research methodology. The fourth part provides data analysis, the fifth part is a discussion, and the sixth part covers the study’s implications. Part VII contains conclusions and future perspectives.

## Theoretical model and research hypotheses

### Theoretical framework

Based on social cognitive theory, which originated from Bandura [25] extension and modification of behaviorist psychology, Wolff, Hilpert [26] emphasized that the three factors – individual cognition, behavior, and environment – dynamically interact with each other and that the three are inextricably intertwined. It provides a new perspective on how personal and environmental factors influence willingness and how to change or adjust one’s behavior [27]. Social cognitive theory is widely used to explain and predict human behavior, especially in educational settings. Social cognitive theory has been applied to examine how interventions can enhance individuals’ self-efficacy and behavioral intentions [28], the influence of the external environment on an individual’s employability [29], analyze the sources of an individual’s self-efficacy [30] and reveal the drivers and mediators in the formation of choice intentions [31]. It examines the drivers and mediators affecting individual choice intention from three dimensions: individual, environment, and behavior. Therefore, it is particularly suitable for studying the factors of teachers’ intention to adopt AIGC to the classroom in higher education, and this study is consistent with the body of well-established literature that provides a comprehensive understanding of the factors that influence teachers’ adoption of AIGC in the classroom. Additionally, examining social cognitive theory enriches the dimensions of the study by providing a more comprehensive framework that helps to improve the predictive accuracy of the model and promotes the successful use of AIGC in the classroom by college teachers. Therefore, this study aims to promote the understanding of intentional factors affecting college teachers’ use of technology based on social cognitive theory, thus providing useful information for educational policy and practice.

### The relationship between self-efficacy and their willingness to choose to adopt AIGC from the perspective of higher education teachers

Self-efficacy is an individual’ s confidence in completing a specific task [32]. In an educational setting, teachers’ self-efficacy is defined as their judgment of their ability to manage the classroom, influence student achievement, and perform assigned tasks [33]. It is considered a prerequisite for teaching motivation and a major driving force for teachers’ teaching work [34,35]. Existing research confirms that Self-efficacy emphasizes teachers’ confidence in teaching, which will affect teachers’ making more informed choices in future teaching [36]. If teachers adopt AIGC to teaching, they will get good returns. When individual outcome expectations meet their expected levels, the teachers’ willingness to choose AIGC to teach. Al-Adwan, Meet [37] supported this idea in a study of higher education teachers’ perceptions of educational technology. Therefore, the following hypotheses are proposed:

Hypothesis 1: Teachers’ willingness to adopt AIGC positively correlates with their respective degrees of self-efficacy.

### The relationship between outcome expectations and self-efficacy and willingness to choose from the perspective of higher education teachers

Outcome expectations are individuals’ predictions of the possible outcomes of engaging in a particular behavior [38]. In general, Individuals with high self-efficacy will predict more positive outcomes. In contrast, individuals with low self-efficacy may predict adverse consequences, which in turn will affect individuals’ willingness to choose [39,40]. Specifically, teachers adopt AIGC into the classroom, and students increase their classroom participation when they are influenced by something new [17], which makes the teacher’ s teaching more emotional, affecting the teacher’ s willingness to adopt AIGC. This highlights that the AIGC supports teachers by allowing them to improve their teaching abilities and strategies. Therefore, based on the available literature, the following hypotheses are proposed:

Hypothesis 2: The outcome expectations of teachers adopting AIGC are expected to play a mediating role in the process of teachers’ self-efficacy and their willingness to adopt AIGC.

### The relationship between self-efficacy enhancement, self-efficacy, and outcome expectations from the perspective of higher education teachers

Self-improvement ability of teachers in higher education refers to the ability of teachers to improve their educational and teaching skills and professionalism through continuous learning and practice. Previous studies have shown that teachers’ outcome expectations are easily influenced by their self-improvement [41]. Teachers with high self-efficacy have more potent learning abilities, are more likely to achieve teaching goals, and work harder [42]. Teachers with high self-efficacy are more likely to establish closer relationships with students and reduce conflict because they are more confident in their ability to improve student classroom participation and use effective strategies in classroom management [43]. When teachers’ self-efficacy is enhanced, they will better understand the advantages of AIGC in the classroom and have expectations of the results of using AIGC. Therefore, the following hypothesis is proposed:

Hypothesis 3: Teachers’ self-efficacy enhancement strengthens the relationship between teachers’ self-efficacy and their expectations of the results of adopting AIGC.

### The relationship between external environmental factors, self-efficacy, willingness to choose, and outcome expectations from the perspective of higher education teachers

This study’s external environmental factors include technical support, industry change, and school support. In the educational environment, existing studies have confirmed that external factors like macro policies and school support will affect teachers’ professional development [44]. Crawley [45] showed that when schools and education departments encourage and support introducing new technologies, teachers will be more inclined to adopt these new technologies. Yuan, Brigandi [45] believes that when schools continuously train teachers to use new technology thinking, it will significantly improve their self-efficacy and enhance their self-confidence, making them more willing to practice in the classroom. The external environmental factors support the view that teachers with high self-efficacy in adopting AIGC are more likely to actively regulate their emotions and teaching abilities. Avşar and Pekmezci [46] believe that when the external environment meets an individual’s needs, their intrinsic motivation is brought to light and triggered, leading to goal-oriented behavior in fulfilling those willings. Therefore, the following hypotheses are proposed:

Hypothesis 4: External environmental factors strengthen the process relationship between teachers’ self-efficacy and their willingness to adopt AIGC.

Hypothesis 5: External environmental factors strengthen the process relationship between teachers’ self-efficacy and their outcome expectations of adopting AIGC.

Hypothesis 6: External environmental factors strengthen the process relationship between teachers’ outcome expectations of adopting AIGC and their willingness to adopt AIGC.

**Fig 1:**
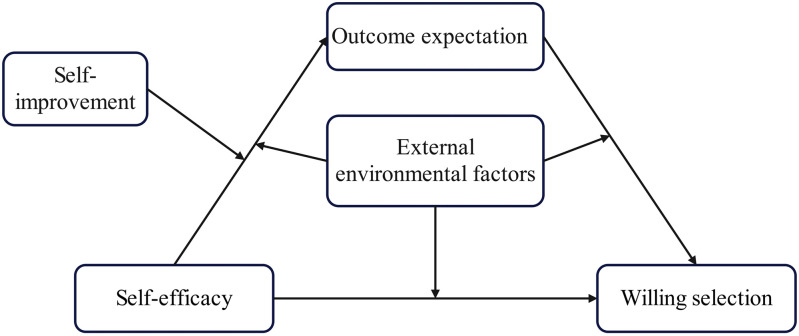
Theoretical model.

As seen in Fig 1, a theoretical model is put out based on these theories.

## Research method

This study used quantitative methods to investigate the willingness of college and university teachers to adopt AIGC to their classroom teaching choices. The data were obtained from a questionnaire survey of teachers in 42 colleges and universities. The questionnaires were administered to college teachers whose age stage is in the range of 22–45 years old and who have been teaching for more than 5 years as the dominant respondents, and the email addresses of the individual teachers were found on their homepages on the official websites of the colleges and universities. Taking these particular respondents as the lead is more conducive to accomplishing the objectives of this study. Therefore, the respondents’ basic information was asked at the beginning of the questionnaire to assess whether or not they were eligible for the survey. Data collection for this study began on March 15, 2024, and ended on April 15, 2024. Ethical approval for this study was obtained from the Academic and Ethics Committee of the College of Architecture and Civil Engineering, University of Western China, before the distribution of the questionnaire. The ethical approval number assigned to this study was No. 12–202304. The authors have duly obtained informed consent from all participants. Before completing the questionnaire, participants were fully informed about the study’s objectives, procedures, and potential implications, as well as the fact that this study had received ethical approval from the Academic and Ethics Committee of the School of Architecture and Civil Engineering, WVU. Subsequently, participants voluntarily provided their signatures indicating their explicit agreement and understanding of the terms and conditions outlined therein. A total of 527 questionnaires were initially received, but after removing outliers incomplete and inconsistent responses, the final valid sample consisted of 513 responses. The sample size was deemed suitable and met the guidelines suggested by Kock and Hadaya [47]. The data cleaning process was performed using Statistical Package for the Social Sciences (SPSS)to ensure the reliability and validity of the collected data. In the questionnaire, the scales of teachers’ self-efficacy in adopting AIGC and teachers’ willingness to choose to adopt AIGC were adapted from Chai, Lin [48]. In the questionnaire, the scales for the three sub-dimensions of external environmental factors (i.e., industry change, technical support, and school support) were adapted from Chou, Shen [49]. The scales for self-improvement ability and outcome expectations of teachers adopting AIGC were adapted from Wang and Chen [50]. Each item contains three items.

The questionnaire consisted of two parts. The first part of the questionnaire began with collecting background information about the respondents, such as gender, age, and years of teaching experience, as shown in Table 1. The second part consisted of a number of items that were used to quantify the variables in the model. The items in the second section were set up with options using a 5-point Likert scale format, ranging from indicating strong disagreement to strong agreement. Jill and McCoach [51] found that compared to a 4-point Likert scale, a 5-point scale provides a neutral option that allows respondents to express their opinions without explicitly agreeing or disagreeing. This allows for their truest attitudes and perceptions and high-quality data results to be obtained, Nemoto and Beglar [52] argued that using a Likert scale to measure different aspects of teaching and learning significantly improves the reliability and validity of the measurements, thus capturing the intended constructs more accurately. The study used this structured questionnaire to quantify university teachers’ intentions to adopt generative AI in teaching and test the hypothesized relationships between the proposed variables. To ensure the quality of the questionnaire, linguistic and educational experts were consulted to evaluate and revise the questionnaire before distributing the formal questionnaire, after which a pre-survey was conducted to optimize the individual questions in the questionnaire to form the final scale.

**Table 1:** Descriptive statistics of the study population.

Form	Categorization	Frequency	Percentage
Distinguishing between the sexes	male	248	48.3%
	women	265	51.7%
(A person’ s) age	22-28 years	49	9.6%
	29-35 years	74	14.4%
	36-42 years	145	28.3%
	43-49 years	177	34.5%
	Over 50 years old	68	13.3%
Length of teaching experience	Less than 7 years	122	23.8%
	8-14 years	175	34.1%
	15-20 years	156	30.4%
	More than 20 years	60	11.7%
Education attainment	undergraduate (adjective)	57	11.1%
	Master’ s degree student	125	24.4%
	PhD student	300	58.5%
	(sth. or sb) else	31	6.0%
Course Type	Specialized Compulsory Courses	90	17.5%
	specialized elective	107	20.9%
	practical course	182	35.5%
	Other courses	134	26.1%

In this study, it is known from reading models of social cognitive theory and related literature that constructing structural equation modeling is the preferred method when analyzing data in social sciences [53,54]. Structural equation modeling focuses on analyzing causal relationships between variables. However, due to the presence of both mediation and moderation in this study, the moderated mediation effect should be tested [55], which is limited in the SmartPLS software because it cannot run moderated mediation analyses on the paths of the model [56]. Instead, Hayes [57] SPSS PROCESS macro (Model 4) and SPSS PROCESS macro (Model 61), a computational tool for mediation and regulation analysis based on path analysis, were integrated into the Conditional Process Model. Since our model consists of one independent variable, two moderating variables, one mediating variable, and one dependent variable, Hayes’ PROCESS macro was chosen as the most appropriate for analyzing the data in this study to gain a deeper and more thorough understanding of the linkages between the variables.

## Results

The data collected were analyzed using SPSS27 software, and the analysis was conducted in two stages – measurement modeling and structural modeling. Firstly the data of this study were analyzed for data validity and reliability using Partial Least Squares (PLS) for the following reasons [58]: because it maximizes the variance explained in the dependent variable rather than theoretical confirmations; the sample size is moderate (n = 513); the research model is complex and involves intermediation and moderation within the hypothesis, and it can also be used to diagnose the relationship between the variables with the multicollinearity problems of variables used in regression equations. Second, to test the mediating role of outcome expectations between self-efficacy and willingness to choose in the perspective of the adoption of AIGC by university teachers, this study used the process macro model4 proposed by Hayes [57] based on SPSS software and set a 95% confidence interval. Finally, to test the mediating role of self-efficacy enhancement and external environmental factors as moderating effects under the perspective of AIGC adoption by university teachers, data were analyzed based on the PROCESS macro model 61 proposed by Hayes [57]. A bootstrap sample of 5000 was specified as well and a 95% confidence interval was set, at which point the composition of the moderating variables (self-competence enhancement and external environmental factors) and the remaining variables (teachers’ self-efficacy, teachers’ outcome expectations of adopting AIGC, and teachers’ willingness to choose to adopt AIGC) influenced the overall structural model in the form of interaction terms, which were analyzed by means of Johnson- Neyman plot, identifying all control variables.

### Measurement model

The reliability, convergent validity, and discriminant validity of the model structure were assessed by evaluating the measurement model.

First, the reliability and convergent validity of the model structure are assessed. In this study, Cronbach’s Alpha coefficient, Composite Reliability (CR), and Average Variance Extraction (AVE) were taken to determine the reliability and convergent validity of the measurement model [58], and the results were shown (see Table 2). The reliability of the total of all dependent variable items was tested, and the Cronbach’s Alpha coefficient was 0.849, indicating that the measurement model was sufficiently internally consistent [58] and the Cronbach’s Alpha coefficient of all the items was greater than 0.700, indicating that all the items had acceptable item reliability. All constructs met the recommended thresholds of CR>;0.7 and AVE>;0.5. Composite Reliability (CR) and Average Variance Extracted (AVE) indicate sufficient internal consistency and convergent validity noted that Hair, Hult [58] states that variable correlations should be less than 0.85 to validate the measurement model. Correlations less than 0.85 are valid because multicollinearity is not an issue.

**Table 2:** Reliability and validity evaluation.

Construct	No. of Items	Cronbach’s Alpha	CR	AVE
self-efficacy	3	0.701	0.78	0.64
Expected results	3	0.804	0.85	0.61
voluntary choice	3	0.843	0.84	0.63
Self-competence enhancement	3	0.800	0.83	0.58
External environmental factors	9	0.801	0.81	0.66

The next step was to assess the discriminant validity of the measurement model, based on the criteria of Fornell and Larcker [59] and the heterozygous trait-monozygous trait (HTMT) correlation ratio criteria [60]. Table 3 shows that the square root of the AVE for each construct (shown on the diagonal) is greater than the relevant inter-construct correlation in the construct correlation matrix [61] and that all HTMT values (above the main diagonal) are below the threshold of 0.85 [60], further demonstrating discriminant validity. The results of the measurement modeling suggest that the scales used in this study are reliable and valid.

**Table 3:** Discriminant validity assessment.

	Mean	SD	SE	OE	WS	SI	EEF
SE	3.468	1.294	0.801	0.725	0.463	0.376	0.343
OE	3.429	1.049	0.632	0.785	0.458	0.383	0.196
WS	3.501	1.375	0.430	0.444	0.796	0.652	0.434
SI	3.549	1.531	0.302	0.309	0.511	0.767	0.175
EEF	3.326	3.387	0.255	0.259	0.311	0.219	0.817

“The criterion on Fornell-Larcker is located below the main diagonal, while the Heterotrait-Monotrait Ratio (HTMT) is positioned above the main diagonal”.

- Main diagonal (in bold) represents √AVE.

### Structural model

After measuring the model to determine the structural model, structural equation modeling was constructed by reading the models of social cognitive theory and related literature. The macro program PROCESS in SPSS27 was used to test the mediating role of outcome expectations, Model4 was used to test whether competence had a moderating effect on the pathway of self-efficacy to outcome expectations, and finally, Model61 was used to test the moderating effect of self-competence enhancement and external environmental factors, and simple slope plots were drawn to analyze the moderating effect.

#### Hypothesis testing.

Path analyses were first conducted without moderators (i.e., increased autonomy, external environmental factors) to test the main and mediating effects of Hypotheses 1 and 2.

Edwards and Lambert [62]bootstrap method from a sample of 5000 was used to test the mediating effect. The mediated effect values and their 95% bootstrap confidence intervals are shown in Table 4; there was a significant positive direct effect of teacher self-efficacy and teachers’ willingness to choose to adopt the AIGC ((direct effect = 0.239, SE = 0.061,95% CI = [0.120,0.359], excluding zeros); the outcome of teacher adoption of the AIGC was expected to be a significant difference between teachers’ self-efficacy There was a significant positive indirect effect between teachers’ self-efficacy and teachers’ willingness to choose to adopt the AIGC ((indirect effect = 0.284, SE = 0.048,95% CI = [0.198,0.386], excluding null). Therefore, hypotheses 1 and 2 are supported.

**Table 4:** Path analysis results for main and mediating effects.

	eff	se	t	p	LLCI	ULCI
direct effect	0.2394	0.0611	3.9206	0.0001	0.1195	0.359
intermediary effect	0.2841	0.0483	0	0.0000	0.1982	0.386
aggregate effect	0.5236	0.0603	8.6837	0.0000	0.4051	0.642

* p < 0.05, **p < 0.01, ***p < 0.001.

**Fig 2:**
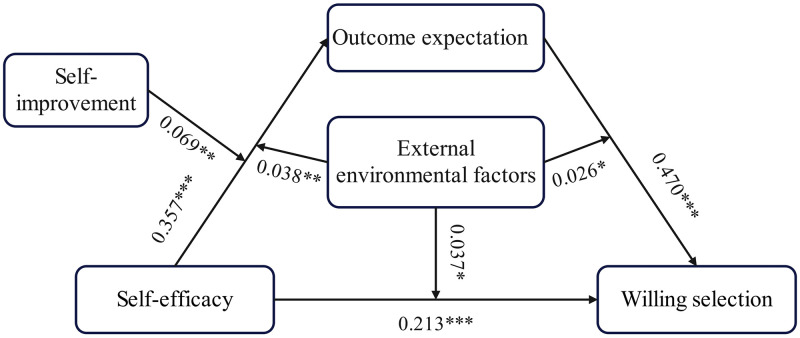
The integrated model.

Subsequently, a path analysis was conducted using PROCESS to test whether the survey factors (i.e., self-efficacy enhancement and external environmental factors) moderated the association between college teachers’ self-efficacy and teachers’ willingness to choose to adopt AIGC. Table 5 and Fig 2 show the results of the moderated analyses and their 95% bootstrap confidence intervals. The regression models showed that the interaction between SE and SI was positively associated with OE (b = 0.069, 0.001 < p < ; 0.01); the interaction between SE and EEF was positively associated with OE (b = 0.038, 0.001 < p < 0.01); the interaction between SE and EEF was positively associated with WS (b = 0.037, 0.01 < p < 0.05); the interaction between OE and EEF was positively correlated with WS (b = 0.026, 0.01 < ; p < 0.05).

**Table 5:** Results of path analysis of moderated and moderated mediated effects.

Predictors	OE			WS		
	b	se	t	b	se	t
SE	0.357	0.064	5.606***	0.213	0.615	3.466***
SI	0.251	0.053	4.780***			
EEF	0.093	0.016	5.755***	0.059	0.020	2.930**
SE x SI	0.069	0.026	2.658**			
SE x EEF	0.038	0.015	2.619**	0.037	0.018	2.028*
OE				0.470	0.048	9.701***
OE x EEF				0.026	0.013	1.978*
*R* ^2^	0.294			0.306		
F	42.204***			44.766***		

Note: SE, self-efficacy; OE, Outcome expectation; WS,Willing selection; SI, self-improvement; EEF,External environmental factors. * p < 0.05, **p < 0.01, ***p < 0.001.

**Fig 3:**
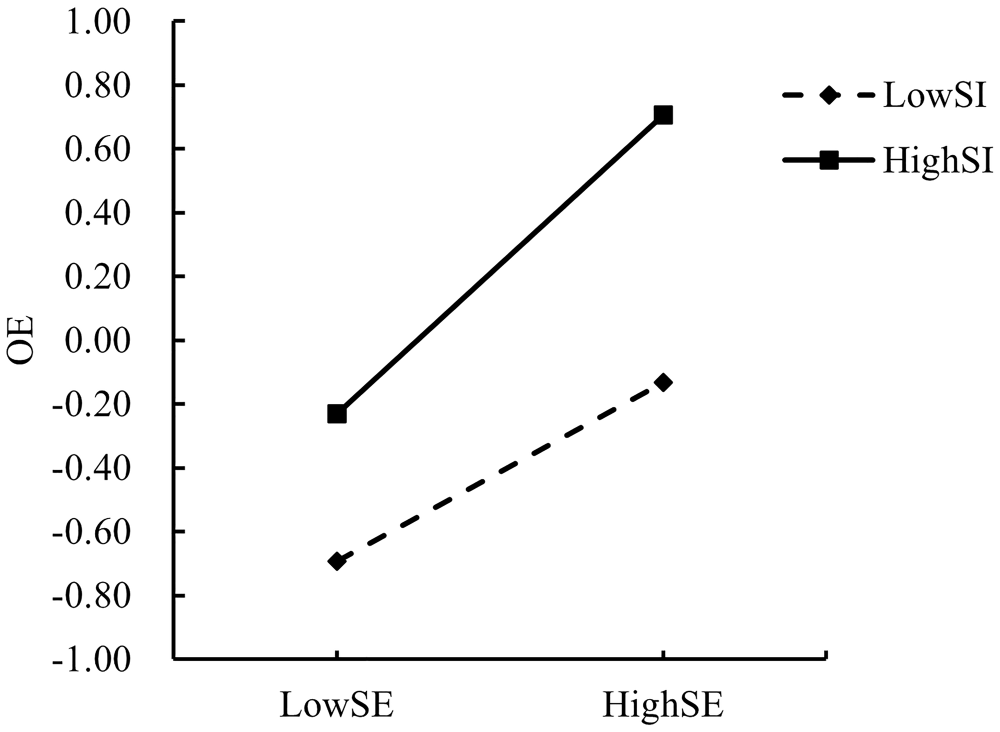
Moderating effect of SI on the relationship between SE and OE.

**Fig 4:**
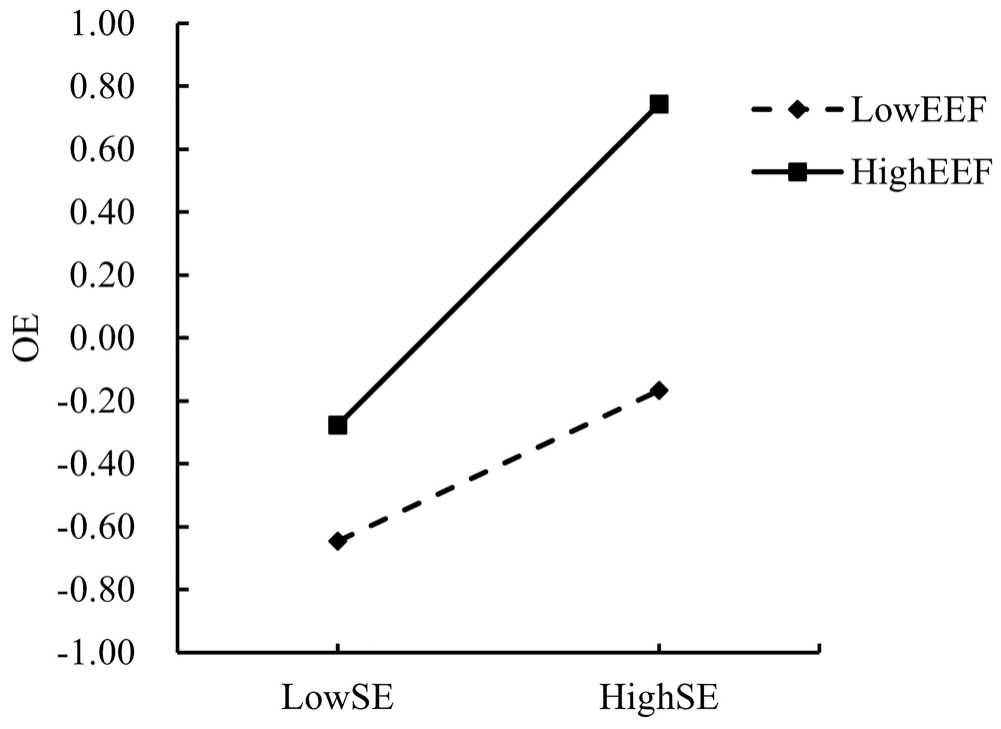
Moderating effect of EEF on the relationship between SE and WS.

**Fig 5:**
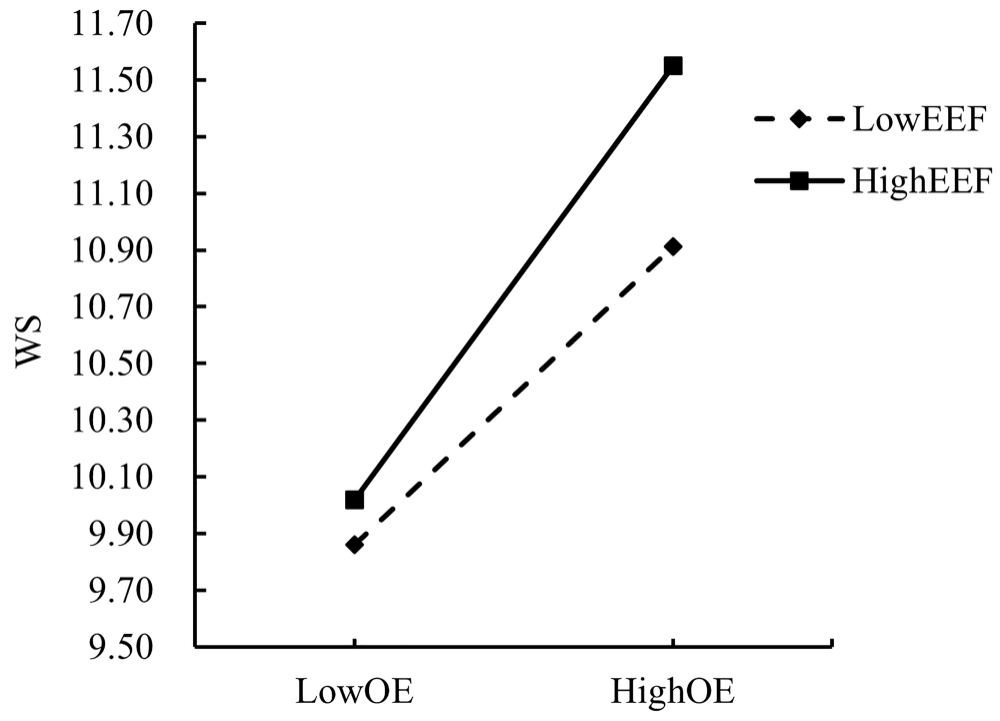
Moderating effect of EEF on the relationship between SE and OE. Hypothesis 6 suggests that external environmental factors strengthen the relationship between the process of outcome expectations of AIGC adoption by college faculty and their willingness to adopt AIGC choices, as indicated by the simple slope plot. Fig 6 shows that when the level of EEF is high (one SD above the mean, conditional effect = 0.557, SE = 0.071, p < 0.001), the positive SE effect on OE is significant and higher than when the level of EEF is low (one SD below the mean, conditional effect = 0.382, SE = 0.060, p < 0.001), and the positive OE effect on OE is significant. The effect was substantial and higher than when the EEF was low (one SD below the mean, conditional effect = 0.382, SE = 0.060, p < 0.001). Therefore, hypothesis 6 is valid.

**Fig 6:**
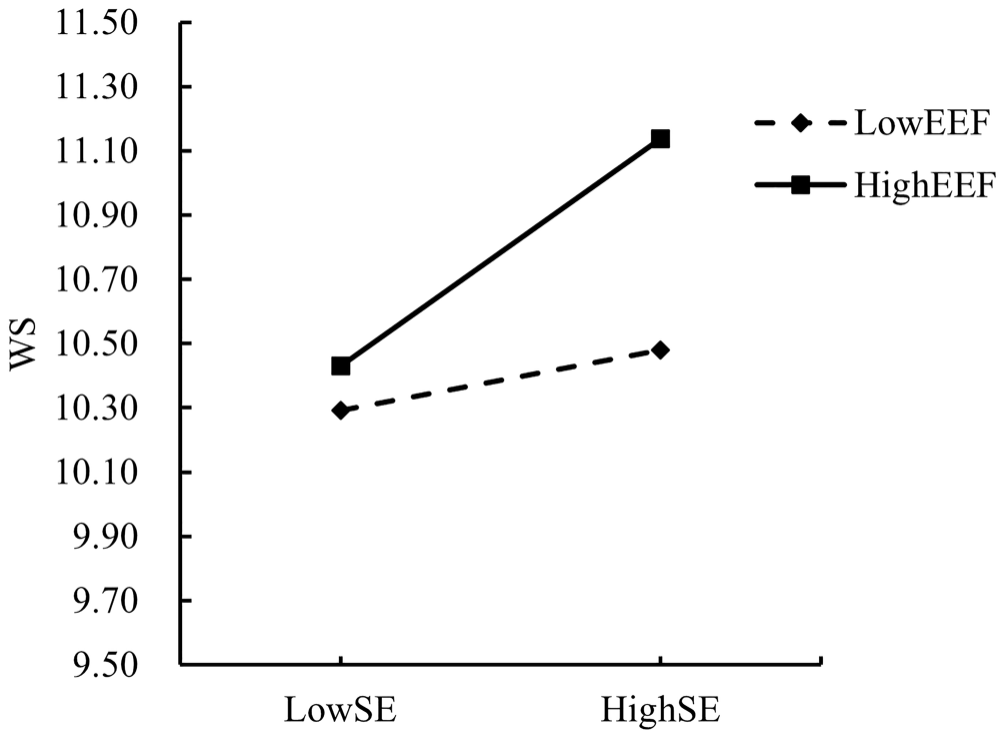
Moderating effect of EEF on the relationship between OE and WS.

In order to explain the moderating effects of SI and EEF more intuitively and clearly, the present study adopted the “mean ± one standard deviation” method according to the previous practice of Dearing and Hamilton [63], setting SI and EEF scores that were one standard deviation above the mean as the high group, and scores that were one standard deviation below the mean as the low group. The moderating effect of EEF on the direct path “SE → WS” and the mediating paths “SE → OE” and “OE → WS” was also confirmed by the moderating effect of SI on the “SE → OE” path and the moderating effect of EEF on the “SE → OE” path. The moderating effects of EEF on the direct path “SE → WS” and the mediating paths “SE → OE” and “OE → WS,” as well as the moderating effects of SI on the path “SE → OE” were analyzed by simple slope analysis plots as shown in Figs 3–6, respectively.

Hypothesis 3 suggests that higher education teachers’ self-efficacy enhancement strengthens the relationship between their self-efficacy and their outcome expectations of adopting the AIGC, as indicated by the simple slope plots in Fig 3, which shows that when the level of SI is high (one SD above the mean, conditional effect = 0.446, SE = 0.075, p < 0.001), the positive impact of SE on OE is significant and that the positive effect of SI on OE is substantial when the level of SE is low (one SD above the mean, conditional effect = 0.446, SE = 0.075, p < 0.001), and higher than when the SI was low (one SD below the mean, conditional effect = 0.268, SE = 0.069, p < 0.001). Therefore, hypothesis 3 is valid.

Hypothesis 4 suggests that external environmental factors strengthen the relationship between the process of self-efficacy of college teachers and their willingness to choose to adopt AIGC, as indicated by the simple slope plot. Fig 4 shows that when the level of EEF is high (one SD above the mean, conditional effect = 0.337, SE = 0.062, p < 0.001), the positive impact of SE on WS is significant However, at low levels of EEF (one SD below the mean, conditional effect = 0.089, SE = 0.082, p > 0.05), the impact of SE on WS became insignificant. Therefore, hypothesis 4 was established.

Hypothesis 5 suggests that external environmental factors strengthen the relationship between the process of self-efficacy of college teachers and their outcome expectations of adopting the AIGC, as indicated by the simple slope plot. Fig 5 shows that when the level of EEF is high (one SD above the mean, conditional effect = 0.486, SE = 0.077, p < 0.001), the positive impact of SE on OE is significant and was higher than when EEF was low (one SD below the mean, conditional effect = 0.228, SE = 0.084, p < 0.01). Therefore, hypothesis 5 is valid.

## Discussion

This study revealed the influence of college teachers’ self-efficacy in adopting generative AI in supporting their willingness to adopt generative AI. The self-efficacy of university teachers in adopting generative AI was found to be highly influential in helping the willingness choice of university teachers to adopt generative AI, indicating that H1 was accepted. This is consistent with extant literature studies [53,64], which implies that teachers with high self-efficacy in adopting generative AI are more likely to accept it and show greater willingness to explore its complex features.

The results showed that college teachers’ self-efficacy in adopting generative AI could influence their choice of willingness to adopt generative AI by influencing teachers’ outcome expectations of adopting generative AI and then influencing their choice of willing to adopt generative AI, which supports H2, which is in line with previous studies [65,66]. This implies that teachers with high-tech self-efficacy will contribute to teachers having a fuller teaching mood and strengthen their willingness to adopt generative AI to bring into their future teaching practice. Therefore, maintaining the outcome expectations of college teachers to adopt generative AI is crucial for promoting generative AI in future teaching practices.

The results of this study provide evidence that teachers’ self-efficacy enhancement significantly impacts the relationship between teachers’ self-efficacy in adopting generative AI and their outcome expectations in adopting generative AI. Therefore, H3 is supported. This is consistent with previous research [67,68]. When teachers perceive insufficient self-efficacy enhancement, even if they have high self-efficacy, they may not be able to achieve these positive outcome expectations due to a lack of competence.

The findings suggest that external environmental factors positively influence the relationship between self-efficacy and willingness to choose, between self-efficacy and outcome expectations of college teachers adopting AIGC, between self-efficacy and outcome expectations of college teachers adopting AIGC, and between outcome expectations and willingness to choose college teachers adopting AIGC in support of H4, H5, and H6, which is consistent with previous studies [69–71]. These studies suggest that in the future, college teachers will be more inclined to integrate generative AI into their future teaching practices if external environmental factors are favorable to generative AI.

## Research significance

### Theoretical significance

First, social cognitive theory is used to identify the drivers that influence college teachers to adopt AIGC to the classroom, providing a new perspective on technology integration education. Second, social cognitive theory was used as a starting point to explore the factors affecting college teachers’ use of AIGC from three dimensions: environmental, personal, and behavioral and this multidimensional analysis helped to comprehensively understand the motivations, barriers, and behavioral patterns of college teachers in the process of adopting AIGC technology. In conclusion, AIGC as an emerging technology has initiated changes in several fields globally, but its decision-making process in higher education scenarios, especially regarding whether college teachers introduce it into classroom teaching or not, has been relatively understudied. This study not only fills the theoretical gap of the application of AIGC in the field of higher education but also provides new ideas and methods for the deep integration and synergistic development of college teachers and AIGC, which helps to promote the development of the education field in the direction of intelligence, personalization, and efficiency.

### Practical significance

The study results suggest that a multidimensional approach is needed to motivate college teachers to adopt generative AI into their classroom teaching. These approaches include encouraging teaching practices and case sharing to enhance college teachers’ self-efficacy. Meanwhile, organizing AI teaching competitions and establishing teacher communities can help improve college teachers’ self-improvement. In addition, focusing on the needs and concerns of college teachers and providing ongoing presentations on new technologies prompts positive outcome expectations. Creating a positive atmosphere and community support promotes communication and collaboration among college teachers through mentoring, sharing, and positive encouragement for mutual improvement. Integrate training, community building, and success story sharing to achieve the goal of widespread adoption and effective integration of AIGC technology into teaching and learning by faculty. Continuous assessment of faculty needs and evaluation of the impact of technology implementation plans can further optimize the use of AIGC technology in teaching and learning. Therefore, the three aspects of improving the self-efficacy and outcome expectations of college teachers in adopting AIGC to classroom teaching and the self-improvement ability of teachers can significantly increase the willingness of college teachers to adopt AIGC technology to classroom teaching choices, thus realizing the transformation and upgrading of the form of classroom teaching in colleges and universities.

In addition, schools need to adopt comprehensive strategies to optimize the conditions of use and provide management support to motivate teachers to integrate AIGC into classroom teaching. Leadership should ensure that the necessary infrastructural support is provided, incentives are created, and a positive culture is fostered to demonstrate the school’s unwavering support for the use of new technologies. Administrators need to regularly assess teachers’ needs in using AIGC technology, examine its effectiveness in teaching practice, and then provide targeted training programs to ensure that college teachers can continue to improve their skills and increase their opportunities for practice.

A deeper understanding of the factors influencing college teachers’ adoption of AIGC in classroom teaching was gained based on social cognitive theory. Specifically, this study illuminates the complex dynamics among college teachers’ self-efficacy to adopt AIGC to the classroom, outcome expectations, willingness to choose, college teachers’ self-efficacy to improve their skills, and external environmental factors. The findings of this paper can provide valuable references for policymakers, educational administrators, and those responsible for teacher technology training to enhance the willingness of college teachers to adopt AIGC technology into the classroom.

### Summarize

This study is based on social cognitive theory, which investigates the factors influencing college teachers’ willingness to choose to apply AIGC technology to classroom teaching. The results of the study show that self-efficacy is a key factor of willingness to choose from the perspective of college teachers; the positive influence of self-efficacy on the willing to choose is more significant when the level of outcome expectation is higher; external environmental factors will strengthen the positive impact of self-efficacy on outcome expectation and willingness to choose; and the ability of self-improvement will also enhance the positive influence of self-efficacy on outcome expectation. The research in this paper provides a more comprehensive understanding of the factors that influence the use of AIGC technology in the classroom and enriches the social cognitive theory in higher education research. The findings suggest that administrators and policymakers should improve teachers’ self-efficacy in adopting AIGC, determining outcome expectations, and improving teachers’ self-improvement abilities to facilitate higher education teachers’ use of AIGC technology in the classroom. The results of this study fill the theoretical gap in the application of AIGC in higher education from the perspective of college teachers and advance the deep integration of college teachers and AIGC technology. The results of this study also contribute to the realization of UNESCO’s Education 2030 Framework for Action, which emphasizes the use of digital technology to achieve universal access and quality improvement in education. It promotes educational equity and facilitates educational innovation, making an essential contribution to the development and progress of global education.

### Limitations and future research directions

This study has some limitations that restrict the generalizability of the findings. First, the data for this study relied on self-reporting by college teachers, which is somewhat subjective. Future studies could select areas with rapid and slow economic development to collect data or collect data from Higher Vocational Education and Regular Higher Education to enhance the robustness and generalizability of the findings. Second, based on this study, it is recommended to incorporate other factors that may influence the willingness choice of higher education teachers to adopt AIGC to the classroom, such as adding AI ethics and regional culture as influencing factors, to use the social cognitive theory model for research in the field of education. Third, compared to the total population, this study has a high percentage of college teachers aged 36–49 years old, and future research could fix the age group or include the age of college teachers as a variable in the model. Overall, this study made a significant contribution to the literature—future research may provide additional insights into the willingness choices of college faculty to adopt AIGC.
